# Supplementary material for: Epidemiological Characterization of Isolates of Salmonella enterica and Shiga Toxin-Producing Escherichia coli from Backyard Production System Animals in the Valparaíso and Metropolitana Regions
Source: Animals (Basel). 2023 Jul 28;13(15):2444. doi: 10.3390/ani13152444 (PMC10417532; doi:10.3390/ani13152444)
Supplement: Supplementary file 1 [file animals-13-02444-s001.zip › Supplementary table 1.pdf]

**Supplementary table 1.** Univariable logistic regression analysis results for *S. enterica*, STEC, and *S. enterica*/STEC models.

| **Dependent variable** | **Independent variables** | **Category** | **OR** | 95% CI | | **p-value** |
|---|---|---|---|---|---|---|
| | | | | **Lower** | **Upper** | |
| *S. enterica* (+) | BPS Positivity to STEC | Positive | 0,000 | | 7,27E+119 | 0,995 |
| | BPS Positivity to *S. enterica*/STEC | Positive | 4,65E+08 | 0,000 | | 0,994 |
| | BPS altitude | - | 0,999 | 0,994 | 1,004 | 0,801 |
| | BPS area (hectares) | - | 0,409 | 0,001 | 1,613 | 0,666 |
| | BPS productive objective | Forestry | 0,000 | | | 0,999 |
| | | Mixed | 0,000 | | 1,56E+178 | 0,996 |
| | | Other | 0,000 | | | 0,998 |
| | | Livestock | 0,767 | 0,088 | 16,346 | 0,826 |
| | BPS productive objective_2 | 2 | 0,000 | | 2,53E+178 | 0,996 |
| | | 3 | 0,000 | | | 0,998 |
| | Economic activity of the household provider | Agricultural worker | 1,79E+08 | 0,000 | | 0,996 |
| | | Dependent employee | 1,000 | 0,000 | 4,63E+218 | 1,000 |
| | | Household and care work | 3,32E+08 | 0,000 | | 0,996 |
| | | Self-employed | 1,000 | 0,000 | 1,11E+209 | 1,000 |
| | Economic activity of the household provider_2 | 2 | 3,51E+07 | 0,000 | | 0,994 |
| | | 3 | 1,22E+08 | 0,000 | | 0,994 |
| | Number of household members | - | 0,964 | 0,535 | 1,271 | 0,866 |
| | Level of importance of animals | 2 | 5,70E+07 | 0,000 | | 0,997 |
| | | 3 | 3,05E+07 | 0,000 | | 0,997 |
| | | 4 | 1,000 | 0,000 | 1,58E+130 | 1,000 |

| | | | | | | |
|---|---|---|---|---|---|---|
| | | 5 | 6,33E+07 | 0,000 | | 0,997 |
| | Bird species raised in the BPS | Mixed | 0,446 | 0,022 | 3,591 | 0,490 |
| | | No | 0,000 | | 8,70E+182 | 0,996 |
| | | Ducks | 0,000 | | 8,70E+182 | 0,996 |
| | Raising pigs at the BPS | Si | 2,479 | 0,119 | 20,732 | 0,444 |
| | Other domestic species raised at the BPS | Goats | 1,000 | 0,000 | | 1,000 |
| | | Rabbits | 1,000 | 0,000 | | 1,000 |
| | | Guinea pigs | 1,000 | 0,000 | | 1,000 |
| | | Mixed | 1,91E+07 | 0,000 | | 0,995 |
| | | Does not apply | 8,38E+06 | 0,000 | | 0,996 |
| | | Sheeps | 1,000 | 0,000 | 2,58E+218 | 1,000 |
| | | Cows | 1,000 | 0,000 | 5,32E+179 | 1,000 |
| | Pets kept at the BPS | No | 3,55E+06 | 0,000 | | 0,994 |
| | | Dogs | 1,18E+06 | 0,000 | | 0,994 |
| | | Dogs and Cats | 5,99E+05 | 0,000 | | 0,995 |
| | Pets kept at the BPS_2 | Yes | 0,216 | 0,025 | 1,874 | 0,135 |
| | Number of gallus gallus raised at BPS | - | 1,006 | 1,001 | 1,012 | 0,023 |
| | Number of waterfowl raised at the BPS | - | 0,884 | 0,442 | 1,037 | 0,519 |
| | Number of other birds raised at the BPS | - | 0,002 | | 1,13E+54 | 0,995 |
| | Number of pigs raised at the BPS | - | 0,975 | | 1,190 | 0,894 |
| | Number of equids raised at the BPS | - | 1,099 | 0,912 | 1,249 | 0,166 |
| | Number of ruminants raised at the BPS | - | 1,000 | | 1,019 | 0,990 |
| | Number of other species raised at the BPS | - | 0,000 | | 9,98E+106 | 0,995 |

| | | | | | | |
|---|---|---|---|---|---|---|
| | Number of pets kept at the BPS | - | 0,529 | 0,164 | 0,935 | 0,135 |
| | Total number of BPS animals | - | 1,005 | 1,001 | 1,010 | 0,013 |
| | Sampling density | - | 0,001 | 0,000 | 4,346 | 0,273 |
| | Gini-Simpson Diversity Index | - | 0,102 | 0,001 | 6,702 | 0,302 |
| | Productive objective of BPS animals | Consumption and sales | 3,88E+07 | 0,000 | | 0,995 |
| | | Hobbies | 1,000 | 0,000 | | 1,000 |
| | | Sales | 1,000 | 0,000 | | 1,000 |
| | Time of rearing of current animals | More than 5 years | 1,000 | 0,000 | 1,47E+210 | 1,000 |
| | | Less than 1 year | 3,52E+07 | 0,000 | | 0,996 |
| | Length of experience raising animals | Between 2 and 10 years | 1,85E+07 | 0,000 | | 0,996 |
| | | More than 20 years | 7,86E+06 | 0,000 | | 0,996 |
| | | Less than 2 years | 1,000 | 0,000 | 3,13E+126 | 1,000 |
| | Gender of the person in charge of animal handling | Male | 1,562 | 0,060 | 40,458 | 0,755 |
| | | Female | 1,887 | 0,175 | 41,343 | 0,609 |
| | Confinement of BPS animals | Mixed | 0,000 | | 7,28E+146 | 0,995 |
| | | Permanent | 1,333 | 0,152 | 28,491 | 0,811 |
| | Variation in the number of animals during the year | No change | 0,189 | 0,015 | 4,470 | 0,203 |
| | | Spring/Summer | 0,065 | 0,002 | 1,801 | 0,066 |
| | Origin of replacement animals | Purchase | 0,000 | | 7,99E+145 | 0,996 |
| | | Their own animals | 0,357 | 0,042 | 3,068 | 0,312 |
| | BPS animal feed | Cereals | 0,000 | | 2,97E+133 | 0,995 |
| | | Mixed | 0,104 | 0,010 | 2,361 | 0,073 |
| | Water source for BPS animals | Mixed | 1,000 | 0,000 | 4,27E+105 | 1,000 |

| | | | | | | |
|---|---|---|---|---|---|---|
| | | Drinking water | 3,94E +06 | 0,0 00 | | 0,995 |
| | | Well water | 3,21E +06 | 0,0 00 | | 0,996 |
| | Treatments applied to sick animals | Mixed | 0,000 | | 5,76E+ 122 | 0,995 |
| | | Nothing | 0,328 | 0,0 15 | 3,534 | 0,369 |
| | | Natural medicines | 0,950 | 0,0 43 | 10,512 | 0,967 |
| | Handling of dead animals | Consumptio n and sales | 1,000 | 0,0 00 | | 1,000 |
| | | Buries them | 3,56E +07 | 0,0 00 | | 0,996 |
| | | Leaves them far from home | 1,000 | 0,0 00 | 1,68E+ 283 | 1,000 |
| | | Mixed | 1,000 | 0,0 00 | | 1,000 |
| | | Nothing | 1,000 | 0,0 00 | 5,63E+ 148 | 1,000 |
| | | Burns them | 1,07E +08 | 0,0 00 | | 0,995 |
| | BPS receives veterinary attention | More than 1 per year | 5,78E +06 | 0,0 00 | | 0,994 |
| | | No | 3,34E +06 | 0,0 00 | | 0,994 |
| | BPS poultry and pigs have contact with each other | Does not apply | 0,124 | 0,0 13 | 2,748 | 0,093 |
| | | Yes | 0,000 | | 2,69E+ 122 | 0,995 |
| | BPS has functional fences | Yes | 1,939 | 0,2 41 | 39,763 | 0,571 |
| | There are watercourses within the BPS | Yes | 0,776 | 0,0 91 | 6,625 | 0,803 |
| | There are watercourses or wetlands near the BPS | Yes | 0,000 | | 5,83E+ 144 | 0,996 |
| | There are neighboring BPSs that raise poultry and swine | Yes | 0,688 | 0,0 81 | 5,869 | 0,712 |
| | There are commercial poultry and swine farms close to the BPS | Yes | 0,000 | | 2,56E+ 102 | 0,994 |
| | BPS birds have free access to watercourses | Yes | 0,429 | 0,0 21 | 3,450 | 0,469 |
| | Backyard animals have contact with wild birds | Yes | 0,065 | 0,0 03 | 0,533 | 0,020 |

| | | | | | | |
|---|---|---|---|---|---|---|
| | BPS animals have contact with neighboring animals | Yes | 0,370 | 0,0 18 | 2,970 | 0,394 |
| | Handling of new animals entering BPS | Does not quarantine | 0,000 | | 7,07E+ 119 | 0,995 |
| | | Quarantines | 0,949 | 0,1 11 | 8,129 | 0,959 |
| | People outside the household have contact with BPS animals | Yes | 3,72E +07 | 0,0 00 | | 0,995 |
| | SAG has visited the BPS | Yes | 1,274 | 0,0 62 | 10,385 | 0,837 |
| | SAG has made a subsequent visit to the BPS | Yes | 8,667 | 0,3 91 | 83,717 | 0,082 |
| | The owner of the BPS is a user of INDAP or PRODESAL | Yes | 0,917 | 0,0 45 | 7,421 | 0,941 |
| | Someone in the family recently had diarrhea recently | Yes | 0,000 | | 2,02E+ 115 | 0,995 |
| | Someone in the family had a fever recently | Yes | 2,881 | 0,1 37 | 24,304 | 0,373 |
| | Anyone in the family had vomiting recently | Yes | 0,000 | | 1,57E+ 134 | 0,995 |
| | Someone in the family recently had poor appetite | Yes | 0,000 | | 5,83E+ 144 | 0,996 |
| | Someone in the family had muscle pain recently | Yes | 1,810 | 0,0 87 | 14,917 | 0,615 |
| | Sick people have contact with the BPS animals | Yes | 3,67E +06 | 0,0 00 | | 0,994 |
| | Pets remain confined indoors | Yes | 0,000 | | 9,16E+ 121 | 0,995 |
| | Pets have access to animal wastes | Yes | 0,266 | 0,0 31 | 5,627 | 0,269 |
| | BPS animals have contact with animals from other BPS | Yes | 0,378 | 0,0 44 | 3,239 | 0,339 |
| | BPS animals have contact with neighbors' pets | Yes | 2,000 | 0,2 34 | 17,113 | 0,495 |
| STEC (+) | BPS Positivity to *S. enterica* | Positive | 0,000 | | 1,67E+ 53 | 0,990 |
| | BPS Positivity to *S. enterica*/STEC | Positive | 1,16E +10 | 0,0 00 | | 0,993 |
| | BPS altitude | - | 1,000 | 0,9 98 | 1,002 | 0,782 |
| | BPS area (hectares) | - | 1,280 | 0,9 69 | 2,007 | 0,139 |
| | BPS productive objective | Forestry | 1,16E +08 | 0,0 00 | | 0,992 |
| | | Mixed | 1,57E +07 | 0,0 00 | | 0,993 |

| | | | | | | |
|---|---|---|---|---|---|---|
| | | Other | 1,000 | 0,000 | 8,68E+30 | 1,000 |
| | | Livestock | 3,63E+07 | 0,000 | | 0,993 |
| | BPS productive objective_2 | 2 | 0,542 | 0,198 | 1,418 | 0,217 |
| | | 3 | 0,000 | | 1,61E+22 | 0,989 |
| | Economic activity of the household provider | Agricultural worker | 1,602 | 0,557 | 4,688 | 0,379 |
| | | Dependent employee | 0,392 | 0,020 | 2,396 | 0,395 |
| | | Household and care work | 0,000 | | 9,33E+34 | 0,991 |
| | | Self-employed | 0,734 | 0,104 | 3,318 | 0,714 |
| | Economic activity of the household provider_2 | 2 | 1,102 | 0,422 | 3,012 | 0,845 |
| | | 3 | 0,000 | | 9,33E+34 | 0,991 |
| | Number of household members | - | 0,902 | 0,686 | 1,096 | 0,393 |
| | Level of importance of animals | 2 | 2,429 | 0,211 | 55,539 | 0,487 |
| | | 3 | 1,962 | 0,229 | 41,480 | 0,573 |
| | | 4 | 2,975 | 0,476 | 57,848 | 0,325 |
| | | 5 | 5,409 | 0,846 | 106,222 | 0,131 |
| | Bird species raised in the BPS | Mixed | 0,770 | 0,269 | 2,058 | 0,609 |
| | | No | 5,500 | 0,208 | 145,659 | 0,239 |
| | | Ducks | 0,000 | | 1,22E+109 | 0,993 |
| | Raising pigs at the BPS | Si | 1,324 | 0,284 | 4,602 | 0,684 |
| | Other domestic species raised at the BPS | Goats | 1,000 | 0,000 | 2,14E+151 | 1,000 |
| | | Rabbits | 1,000 | 0,000 | 9,05E+47 | 1,000 |
| | | Guinea pigs | 1,000 | 0,000 | 3,87E+134 | 1,000 |

| | | | | | | |
|---|---|---|---|---|---|---|
| | | Mixed | 7,71E +07 | 0,0 00 | | 0,992 |
| | | Does not apply | 8,03E +06 | 0,0 00 | | 0,993 |
| | | Sheeps | 5,78E +07 | 0,0 00 | | 0,992 |
| | | Cows | 1,000 | 0,0 00 | 1,87E+ 39 | 1,000 |
| | Pets kept at the BPS | No | 0,391 | 0,0 34 | 9,299 | 0,473 |
| | | Dogs | 0,469 | 0,0 48 | 10,503 | 0,545 |
| | | Dogs and Cats | 0,541 | 0,0 62 | 11,485 | 0,609 |
| | Pets kept at the BPS_2 | Yes | 1,358 | 0,4 12 | 6,159 | 0,647 |
| | Number of gallus gallus raised at BPS | - | 1,002 | 0,9 97 | 1,006 | 0,392 |
| | Number of waterfowl raised at the BPS | - | 0,980 | 0,8 94 | 1,027 | 0,562 |
| | Number of other birds raised at the BPS | - | 1,044 | 1,0 00 | 1,131 | 0,159 |
| | Number of pigs raised at the BPS | - | 1,051 | 0,9 17 | 1,170 | 0,375 |
| | Number of equids raised at the BPS | - | 1,104 | 0,9 98 | 1,238 | 0,057 |
| | Number of ruminants raised at the BPS | - | 1,048 | 1,0 16 | 1,088 | 0,007 |
| | Number of other species raised at the BPS | - | 1,125 | 1,0 15 | 1,331 | 0,124 |
| | Number of pets kept at the BPS | - | 0,978 | 0,8 76 | 1,070 | 0,657 |
| | Total number of BPS animals | - | 1,003 | 1,0 00 | 1,007 | 0,092 |
| | Sampling density | - | 0,544 | 0,0 27 | 4,716 | 0,635 |
| | Gini-Simpson Diversity Index | - | 2,677 | 0,3 69 | 21,800 | 0,339 |
| | Productive objective of BPS animals | Consumptio n and sales | 2,596 | 0,8 02 | 11,660 | 0,148 |
| | | Hobbies | 0,000 | | 2,91E+ 36 | 0,993 |
| | | Sales | 5,25E +08 | 0,0 00 | | 0,996 |
| | Time of rearing of current animals | More than 5 years | 0,000 | | 3,05E+ 35 | 0,992 |

| | | | | | | |
|---|---|---|---|---|---|---|
| | | Less than 1 year | 1,956 | 0,602 | 8,809 | 0,311 |
| | Length of experience raising animals | Between 2 and 10 years | 0,455 | 0,066 | 3,812 | 0,422 |
| | | More than 20 years | 1,029 | 0,237 | 7,172 | 0,972 |
| | | Less than 2 years | 0,625 | 0,026 | 7,732 | 0,720 |
| | Gender of the person in charge of animal handling | Male | 2,098 | 0,515 | 9,102 | 0,298 |
| | | Female | 2,937 | 0,928 | 11,224 | 0,082 |
| | Confinement of BPS animals | Mixed | 0,476 | 0,124 | 2,341 | 0,307 |
| | | Permanent | 0,667 | 0,136 | 3,738 | 0,621 |
| | Variation in the number of animals during the year | No change | 0,729 | 0,097 | 15,039 | 0,787 |
| | | Spring/Summer | 0,909 | 0,131 | 18,226 | 0,933 |
| | Origin of replacement animals | Purchase | 0,000 | | 6,24E+108 | 0,993 |
| | | Their own animals | 0,494 | 0,185 | 1,371 | 0,162 |
| | BPS animal feed | Cereals | 0,267 | 0,009 | 7,738 | 0,385 |
| | | Mixed | 0,720 | 0,099 | 14,516 | 0,775 |
| | Water source for BPS animals | Mixed | 1,333 | 0,104 | 17,648 | 0,819 |
| | | Drinking water | 0,275 | 0,047 | 2,147 | 0,162 |
| | | Well water | 0,313 | 0,046 | 2,669 | 0,240 |
| | Treatments applied to sick animals | Mixed | 0,884 | 0,173 | 3,655 | 0,870 |
| | | Nothing | 0,635 | 0,200 | 2,011 | 0,432 |
| | | Natural medicines | 0,786 | 0,155 | 3,214 | 0,748 |
| | Handling of dead animals | Consumption and sales | 0,000 | | 2,92E+108 | 0,993 |
| | | Buries them | 2,066 | 0,612 | 9,488 | 0,284 |

| | | | | | | |
|---|---|---|---|---|---|---|
| | | Leaves them far from home | 1,500 | 0,068 | 14,318 | 0,744 |
| | | Mixed | 0,000 | | 5,87E+205 | 0,995 |
| | | Nothing | 0,643 | 0,030 | 5,563 | 0,713 |
| | | Burns them | 1,125 | 0,052 | 10,277 | 0,923 |
| | BPS receives veterinary attention | More than 1 per year | 2,13E+07 | 0,000 | | 0,989 |
| | | No | 5,88E+06 | 0,000 | | 0,990 |
| | BPS poultry and pigs have contact with each other | Does not apply | 7,22E+06 | 0,000 | | 0,992 |
| | | Yes | 1,70E+07 | 0,000 | | 0,992 |
| | BPS has functional fences | Yes | 0,587 | 0,224 | 1,536 | 0,272 |
| | There are watercourses within the BPS | Yes | 0,949 | 0,366 | 2,515 | 0,914 |
| | There are watercourses or wetlands near the BPS | Yes | 1,733 | 0,365 | 6,255 | 0,433 |
| | There are neighboring BPSs that raise poultry and swine | Yes | 1,750 | 0,653 | 5,228 | 0,284 |
| | There are commercial poultry and swine farms close to the BPS | Yes | 2,824 | 0,567 | 11,279 | 0,159 |
| | BPS birds have free access to watercourses | Yes | 0,859 | 0,316 | 2,231 | 0,758 |
| | Backyard animals have contact with wild birds | Yes | 4,800 | 0,922 | 88,362 | 0,136 |
| | BPS animals have contact with neighboring animals | Yes | 1,164 | 0,446 | 3,037 | 0,754 |
| | Handling of new animals entering BPS | Does not quarantine | 0,329 | 0,017 | 1,972 | 0,310 |
| | | Quarantines | 1,375 | 0,513 | 3,825 | 0,529 |
| | People outside the household have contact with BPS animals | Yes | 1,053 | 0,389 | 3,168 | 0,922 |
| | SAG has visited the BPS | Yes | 4,050 | 1,462 | 11,113 | 0,006 |
| | SAG has made a subsequent visit to the BPS | Yes | 6,824 | 1,182 | 39,581 | 0,025 |
| | The owner of the BPS is a user of INDAP or PRODESAL | Yes | 2,659 | 0,982 | 7,089 | 0,050 |

| | | | | | | |
|---|---|---|---|---|---|---|
| | Someone in the family recently had diarrhea recently | Yes | 0,842 | 0,044 | 5,123 | 0,876 |
| | Someone in the family had a fever recently | Yes | 0,906 | 0,135 | 3,656 | 0,902 |
| | Anyone in the family had vomiting recently | Yes | 0,000 | | 1,72E+60 | 0,992 |
| | Someone in the family recently had poor appetite | Yes | 0,991 | 0,146 | 4,044 | 0,991 |
| | Someone in the family had muscle pain recently | Yes | 2,000 | 0,590 | 5,959 | 0,231 |
| | Sick people have contact with the BPS animals | Yes | 1,369 | 0,232 | 26,142 | 0,773 |
| | Pets remain confined indoors | Yes | 0,000 | | 9,68E+71 | 0,991 |
| | Pets have access to animal wastes | Yes | 1,935 | 0,345 | 36,387 | 0,538 |
| | BPS animals have contact with animals from other BPS | Yes | 1,667 | 0,564 | 6,132 | 0,390 |
| | BPS animals have contact with neighbors' pets | Yes | 2,216 | 0,842 | 5,853 | 0,104 |
| *S. enterica*/STEC (+) | BPS Positivity to *S. enterica* | Positive | 9,00E+07 | 0,000 | | 0,988 |
| | BPS Positivity to STEC | Positive | 9,04E+09 | 0,000 | | 0,992 |
| | BPS altitude | - | 1,000 | 0,998 | 1,002 | 0,884 |
| | BPS area (hectares) | - | 1,238 | 0,944 | 1,883 | 0,169 |
| | BPS productive objective | Forestry | 11,000 | 0,291 | 573,745 | 0,173 |
| | | Mixed | 1,492 | 0,237 | 29,085 | 0,719 |
| | | Other | 0,000 | | 1,05E+21 | 0,989 |
| | | Livestock | 4,812 | 0,813 | 92,253 | 0,150 |
| | BPS productive objective_2 | 2 | 0,373 | 0,140 | 0,927 | 0,038 |
| | | 3 | 0,000 | | 7,62E+27 | 0,988 |
| | Economic activity of the household provider | Agricultural worker | 2,350 | 0,871 | 6,645 | 0,096 |
| | | Dependent employee | 0,392 | 0,020 | 2,396 | 0,395 |

| | | | | | | |
|---|---|---|---|---|---|---|
| | | Household and care work | 0,839 | 0,0 42 | 5,707 | 0,877 |
| | | Self- employed | 0,734 | 0,1 04 | 3,318 | 0,714 |
| | Economic activity of the household provider_2 | 2 | 1,445 | 0,5 77 | 3,854 | 0,442 |
| | | 3 | 0,839 | 0,0 42 | 5,707 | 0,877 |
| | Number of household members | - | 0,910 | 0,7 12 | 1,089 | 0,388 |
| | Level of importance of animals | 2 | 3,923 | 0,4 44 | 84,537 | 0,259 |
| | | 3 | 2,720 | 0,3 63 | 55,698 | 0,389 |
| | | 4 | 2,975 | 0,4 76 | 57,848 | 0,325 |
| | | 5 | 7,650 | 1,2 49 | 148,48 0 | 0,065 |
| | Bird species raised in the BPS | Mixed | 0,686 | 0,2 58 | 1,712 | 0,429 |
| | | No | 4,200 | 0,1 60 | 110,38 0 | 0,320 |
| | | Ducks | 0,000 | | 9,12E+ 63 | 0,989 |
| | Raising pigs at the BPS | Si | 1,569 | 0,4 10 | 4,978 | 0,469 |
| | Other domestic species raised at the BPS | Goats | 1,000 | | | 1,000 |
| | | Rabbits | 1,000 | 0,0 00 | 1,25E+ 44 | 1,000 |
| | | Guinea pigs | 1,000 | 0,0 00 | 3,70E+ 76 | 1,000 |
| | | Mixed | 9,74E +07 | 0,0 00 | | 0,992 |
| | | Does not apply | 1,16E +07 | 0,0 00 | | 0,993 |
| | | Sheeps | 5,78E +07 | 0,0 00 | | 0,992 |
| | | Cows | 1,000 | 0,0 00 | 6,00E+ 35 | 1,000 |
| | Pets kept at the BPS | No | 0,714 | 0,0 72 | 16,131 | 0,789 |
| | | Dogs | 0,581 | 0,0 61 | 12,837 | 0,661 |

| | | | | | | |
|---|---|---|---|---|---|---|
| | | Dogs and Cats | 0,600 | 0,070 | 12,696 | 0,670 |
| | Pets kept at the BPS_2 | Yes | 0,849 | 0,301 | 2,787 | 0,769 |
| | Number of gallus gallus raised at BPS | - | 1,007 | 1,001 | 1,016 | 0,076 |
| | Number of waterfowl raised at the BPS | - | 0,972 | 0,886 | 1,021 | 0,428 |
| | Number of other birds raised at the BPS | - | 1,037 | 0,995 | 1,115 | 0,173 |
| | Number of pigs raised at the BPS | - | 1,044 | 0,914 | 1,160 | 0,439 |
| | Number of equids raised at the BPS | - | 1,135 | 1,025 | 1,287 | 0,026 |
| | Number of ruminants raised at the BPS | - | 1,048 | 1,016 | 1,088 | 0,008 |
| | Number of other species raised at the BPS | - | 1,106 | 1,007 | 1,300 | 0,153 |
| | Number of pets kept at the BPS | - | 0,944 | 0,842 | 1,034 | 0,264 |
| | Total number of BPS animals | - | 1,008 | 1,003 | 1,017 | 0,027 |
| | Sampling density | - | 0,313 | 0,015 | 2,869 | 0,380 |
| | Gini-Simpson Diversity Index | - | 1,484 | 0,239 | 9,723 | 0,673 |
| | Productive objective of BPS animals | Consumption and sales | 3,426 | 1,085 | 15,205 | 0,059 |
| | | Hobbies | 0,000 | | 1,70E+33 | 0,993 |
| | | Sales | 5,25E+08 | 0,000 | | 0,996 |
| | Time of rearing of current animals | More than 5 years | 0,000 | | 2,99E+32 | 0,992 |
| | | Less than 1 year | 2,538 | 0,798 | 11,305 | 0,155 |
| | Length of experience raising animals | Between 2 and 10 years | 0,806 | 0,147 | 6,236 | 0,814 |
| | | More than 20 years | 1,212 | 0,283 | 8,391 | 0,815 |
| | | Less than 2 years | 0,625 | 0,026 | 7,732 | 0,720 |
| | Gender of the person in charge of animal handling | Male | 2,044 | 0,565 | 7,712 | 0,273 |
| | | Female | 2,848 | 0,983 | 9,498 | 0,065 |

| | | | | | | |
|---|---|---|---|---|---|---|
| | Confinement of BPS animals | Mixed | 0,321 | 0,089 | 1,330 | 0,093 |
| | | Permanent | 0,818 | 0,200 | 3,688 | 0,783 |
| | Variation in the number of animals during the year | No change | 0,391 | 0,065 | 3,122 | 0,318 |
| | | Spring/Summer | 0,400 | 0,070 | 3,098 | 0,318 |
| | Origin of replacement animals | Purchase | 0,000 | | 5,16E+63 | 0,989 |
| | | Their own animals | 0,440 | 0,176 | 1,124 | 0,080 |
| | BPS animal feed | Cereals | 0,100 | 0,004 | 1,358 | 0,095 |
| | | Mixed | 0,325 | 0,051 | 2,579 | 0,234 |
| | Water source for BPS animals | Mixed | 1,333 | 0,104 | 17,648 | 0,819 |
| | | Drinking water | 0,364 | 0,064 | 2,800 | 0,268 |
| | | Well water | 0,387 | 0,059 | 3,237 | 0,330 |
| | Treatments applied to sick animals | Mixed | 0,646 | 0,130 | 2,522 | 0,552 |
| | | Nothing | 0,540 | 0,185 | 1,554 | 0,251 |
| | | Natural medicines | 0,810 | 0,196 | 2,902 | 0,755 |
| | Handling of dead animals | Consumption and sales | 0,000 | | 2,92E+108 | 0,993 |
| | | Buries them | 2,638 | 0,800 | 11,979 | 0,147 |
| | | Leaves them far from home | 1,500 | 0,068 | 14,318 | 0,744 |
| | | Mixed | 0,000 | | 5,87E+205 | 0,995 |
| | | Nothing | 0,643 | 0,030 | 5,563 | 0,713 |
| | | Burns them | 2,571 | 0,296 | 18,709 | 0,348 |
| | BPS receives veterinary attention | More than 1 per year | 2,62E+07 | 0,000 | | 0,989 |
| | | No | 7,48E+06 | 0,000 | | 0,989 |

| | | | | | | |
|---|---|---|---|---|---|---|
| | BPS poultry and pigs have contact with each other | Does not apply | 1,019 | 0,154 | 20,061 | 0,986 |
| | | Yes | 2,000 | 0,143 | 51,833 | 0,615 |
| | BPS has functional fences | Yes | 0,706 | 0,290 | 1,740 | 0,442 |
| | There are watercourses within the BPS | Yes | 0,909 | 0,375 | 2,234 | 0,833 |
| | There are watercourses or wetlands near the BPS | Yes | 1,351 | 0,288 | 4,782 | 0,665 |
| | There are neighboring BPSs that raise poultry and swine | Yes | 1,485 | 0,602 | 3,919 | 0,403 |
| | There are commercial poultry and swine farms close to the BPS | Yes | 2,204 | 0,448 | 8,654 | 0,279 |
| | BPS birds have free access to watercourses | Yes | 0,753 | 0,295 | 1,834 | 0,539 |
| | Backyard animals have contact with wild birds | Yes | 1,117 | 0,374 | 4,137 | 0,853 |
| | BPS animals have contact with neighboring animals | Yes | 0,956 | 0,389 | 2,313 | 0,920 |
| | Handling of new animals entering BPS | Does not quarantine | 0,253 | 0,013 | 1,458 | 0,204 |
| | | Quarantines | 1,300 | 0,522 | 3,320 | 0,575 |
| | People outside the household have contact with BPS animals | Yes | 1,423 | 0,546 | 4,191 | 0,491 |
| | SAG has visited the BPS | Yes | 3,609 | 1,376 | 9,339 | 0,008 |
| | SAG has made a subsequent visit to the BPS | Yes | 11,300 | 2,067 | 85,527 | 0,007 |
| | The owner of the BPS is a user of INDAP or PRODESAL | Yes | 2,328 | 0,911 | 5,820 | 0,071 |
| | Someone in the family recently had diarrhea recently | Yes | 0,671 | 0,035 | 4,034 | 0,715 |
| | Someone in the family had a fever recently | Yes | 1,226 | 0,263 | 4,277 | 0,767 |
| | Anyone in the family had vomiting recently | Yes | 0,000 | | 1,48E+60 | 0,992 |
| | Someone in the family recently had poor appetite | Yes | 0,780 | 0,116 | 3,133 | 0,756 |
| | Someone in the family had muscle pain recently | Yes | 2,063 | 0,668 | 5,795 | 0,182 |
| | Sick people have contact with the BPS animals | Yes | 1,720 | 0,294 | 32,695 | 0,617 |

| | | | | | | |
|---|---|---|---|---|---|---|
| | Pets remain confined indoors | Yes | 0,000 | | 8,05E+71 | 0,991 |
| | Pets have access to animal wastes | Yes | 1,048 | 0,253 | 7,124 | 0,954 |
| | BPS animals have contact with animals from other BPS | Yes | 1,207 | 0,460 | 3,570 | 0,714 |
| | BPS animals have contact with neighbors' pets | Yes | 2,286 | 0,930 | 5,640 | 0,070 |
