# Supplementary material for: Epidemiological Characterization of Isolates of Salmonella enterica and Shiga Toxin-Producing Escherichia coli from Backyard Production System Animals in the Valparaíso and Metropolitana Regions
Source: Animals (Basel). 2023 Jul 28;13(15):2444. doi: 10.3390/ani13152444 (PMC10417532; doi:10.3390/ani13152444)
Supplement: Supplementary file 1 [file animals-13-02444-s001.zip › Supplementary table 2.pdf]

Supplementary table 2. Positive BPS samples, by recovery state, animal species, region, and BPS-ID.

**BPS ID**	**Pathogen**	**Animal species**	**Region**	**Recovered?**
ME001	STEC	Cow	Metropolitana	Yes
ME001	STEC	Cow	Metropolitana	No
ME001	STEC	Cow	Metropolitana	Yes [*]
ME001	STEC	Swine	Metropolitana	No
ME010	STEC	Duck	Metropolitana	No
ME010	STEC	Duck	Metropolitana	No
ME010	STEC	Duck	Metropolitana	No
ME011	STEC	Chicken	Metropolitana	No
ME011	STEC	Sheep	Metropolitana	Yes [ç]
ME024	STEC	Sheep	Metropolitana	No
CORD001	STEC	Cow	Metropolitana	No
CORD003	STEC	Sheep	Metropolitana	No
CORD004	STEC	Sheep	Metropolitana	No
CORD004	STEC	Goat	Metropolitana	No
MAI009	STEC	Chicken	Metropolitana	No
CHAC003	STEC	Sheep	Metropolitana	Yes [+]
CHAC003	STEC	Sheep	Metropolitana	Yes [*]
CHAC003	STEC	Cow	Metropolitana	Yes
CHAC010	STEC	Goat	Metropolitana	No
CHAC010	STEC	Sheep	Metropolitana	No
SF011	STEC	Chicken	Valparaíso	No
SF011	STEC	Goose	Valparaíso	No
SF015	STEC	Sheep	Valparaíso	No
SF017	STEC	Sheep	Valparaíso	No
SF017	STEC	Goat	Valparaíso	Yes
SF019	STEC	Goat	Valparaíso	No
SF021	STEC	Chicken	Valparaíso	No
SF021	STEC	Chicken	Valparaíso	No
SF022	STEC	Chicken	Valparaíso	No
SA001	STEC	Goat	Valparaíso	No
SA001	STEC	Goat	Valparaíso	No
SA001	STEC	Cow	Valparaíso	No
SA007	STEC	Chicken	Valparaíso	No
SA010	STEC	Chicken	Valparaíso	No
SA010	STEC	Chicken	Valparaíso	No
PET003	STEC	Chicken	Valparaíso	No

PET003	STEC	Cow	Valparaíso	No
ME023	*S. enterica*	Chicken	Metropolitana	Yes
ME023	*S. enterica*	Goose	Metropolitana	Yes
ME033	*S. enterica*	Chicken	Metropolitana	Yes
CORD002	*S. enterica*	Chicken	Metropolitana	Yes
MAI013	*S. enterica*	Chicken	Metropolitana	Yes

[*] two isolates recovered; [+] three isolates recovered; [ç] four isolates recovered
